# Dimensional Analysis Using Toric Ideals: Primitive Invariants

**DOI:** 10.1371/journal.pone.0112827

**Published:** 2014-12-01

**Authors:** Mark A. Atherton, Ronald A. Bates, Henry P. Wynn

**Affiliations:** 1 Department of Mechanical Engineering, Brunel University, London, United Kingdom; 2 Department of Statistics, London School of Economics, London, United Kingdom; Universita' del Piemonte Orientale, Italy

## Abstract

Classical dimensional analysis in its original form starts by expressing the units for derived quantities, such as force, in terms of power products of basic units 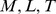 etc. This suggests the use of toric ideal theory from algebraic geometry. Within this the Graver basis provides a unique primitive basis in a well-defined sense, which typically has more terms than the standard Buckingham approach. Some textbook examples are revisited and the full set of primitive invariants found. First, a worked example based on convection is introduced to recall the Buckingham method, but using computer algebra to obtain an integer 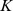 matrix from the initial integer 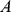 matrix holding the exponents for the derived quantities. The 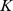 matrix defines the dimensionless variables. But, rather than this integer linear algebra approach it is shown how, by staying with the power product representation, the full set of invariants (dimensionless groups) is obtained directly from the toric ideal defined by 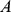. One candidate for the set of invariants is a simple basis of the toric ideal. This, although larger than the rank of 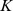, is typically not unique. However, the alternative Graver basis is unique and defines a maximal set of invariants, which are primitive in a simple sense. In addition to the running example four examples are taken from: a windmill, convection, electrodynamics and the hydrogen atom. The method reveals some named invariants. A selection of computer algebra packages is used to show the considerable ease with which both a simple basis and a Graver basis can be found.

## Introduction

Dimensional analysis has a long history. It was discussed by Newton and provided useful intuition to Maxwell, see [12], chapter 3. A recent paper giving popular overview is [21]. The first rigorous and most well-known treatment is by Buckingham [4]–[6], whose name is attached to the main theorem. Dimensional analysis is still considered a fundamental part of physics and is usually taught at an early stage. It is sometimes studied under a heading of qualitative physics [3] [26]. In engineering it gives a useful additional tool for the analysis of systems [15]. It is used in engineering design and in experimental design for engineering experiments [14] [16] [26] and a recent paper with discussion [1] [13]. It has also been used in economics [2]. For an interesting recent application to turbulence and criticality see [7] [8].

We give an algebraic development of dimensional analysis based on the theory of toric ideals and toric varieties. Although this is essentially a reformulation, the algebraic theory itself is by no means elementary and the theory of toric ideals is a live branch of algebraic geometry. We have used [25] and the recent comprehensive volume [11]. We shall see that the methods give all primitive invariants for a particular problem, in a well-defined sense, which are typically more than given by the Buckingham method.

Within mathematical physics dimensional analysis can also be seen as an elementary application of the theory of Lie groups and invariants, when the group is the scale group defined by multiplication. We shall draw on [23] in Section 5.

### 1.1 Basic dimensional analysis

The basic idea of dimensional analysis is that physical systems use fundamental quantities, or units, of mass (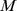), length (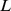) and time (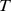). To this list may be added various others such as temperature (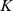) and current (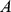), depending on the domain. The extent to which derived quantities can be expressed in terms of 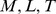 goes to the heart of physics but we shall not delve deeply. Mathematical models for physical systems use so-called *derived* quantities such as: force, energy, momentum, capacity etc. Dimensional analysis tells us that each one of these quantities has units which have a power product representation. Below are a few examples from mechanics.

momentum 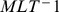


force 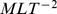


work 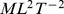


energy 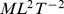


pressure 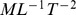


density 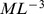


volumetric flow 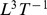


We note that the formulae for the expression of derived units have integer powers. This is critical for our development: it makes them *algebraic* in the sense of polynomial algebra.

In a physical system we may be interested in a special collection of derived quantities. The task of dimensional analysis is to derive dimensionless variables with a view to finding, by additional theory or experiment, or both, the relationship between these dimensionless variables. As mentioned, the key theorem in the area is due to Buckingham. In this section we explain it with an example.

Rather than use the 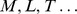 notation we assume that there are some basic quantities of interest which we label 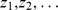. Each such quantity is assumed to have the scaling property, namely if the fundamental units, which we now call 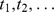 are scaled up or down this induces a transformation on the 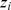. Whether this means simply a change in units or actual physical scaling of the system is sometimes unclear in the literature, but we shall prefer the latter interpretation. Thus the area 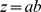 of a rectangle with sides of length 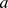 and 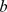, and which would have units 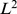, would be transformed into 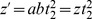. under a physical scaling, 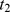, of the length. Although the same scaling would occur with a change of units.

As a another example if 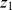 is force and the fundamental units are mass 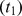, length 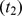 and time 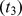, then the scaling transformation is
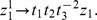



We use the arrow to indicate that scaling of the fundamental quantities 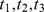 implies a scaling of the derived quantity. With a collection of derived quantities we have one such transformation for each 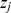. Dimensionless quantities are rational functions of derived quantities for which there is *no* scaling: 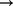 is the identity.

Here is a well known example which we shall use as our running example. It concerns a body in an incompressible fluid and the derived quantities of interest are fluid density (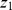), fluid velocity (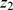), object diameter (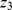), fluid dynamic viscosity (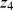) and fluid resistance (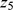) (drag force). Taking the units into account the transformation is:
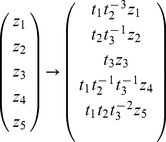
(1)


After a little algebra, or formal use of Buckingham's theorem, we can derive dimensionaless quantities
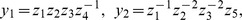
and it is straightforward to check that in each case the 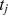 cancel so there is no scaling. The first quantity is Reynolds number and the second is sometimes referred to as the dimensional drag.

To repeat, the term *dimensionless* is interpreted by saying that replacing each 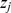 by the 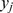 in the transformation 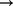 in (1), leaves the 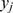 unchanged: they are rational invariants of the transformation.

The dimensionless principal, for our example, embodied in the Buckingham theorem is that *any* function 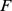 of 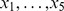 which is invariant under 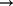 is a function of 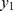 and 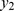 which we write: 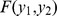.

We now sketch the traditional method. The transformation in (1) can be coded up by capturing the exponents in the power products. This gives
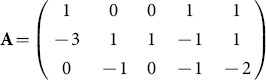



This matrix has rank 3 and we can find a full rank 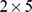 integer kernel matrix, namely an integer matrix 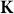 which has rank 2 and such that 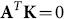. This is readily computed using existing functions in computer algebra, as oppose to a numerical package which may, for example, give a non-integer orthonormal kernel basis. The nullspace or kernal commands in Maple can be used. For the above 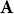 we obtained using these commands
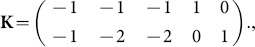
and with a sign change in the first row we obtain



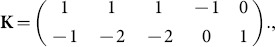



The key point is that the rows of 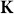 give the exponents of 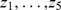 in 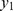 and 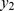, above.

However, because 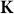 is not unique, because we can choose different bases for the kernel, we could use
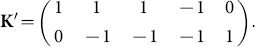



This gives an alternative to 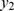, above, namely 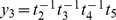. The approach of this paper clarifies, among other issues, the problem of the choice of 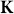 which this example exposes.

## Power Products and Toric Ideals

Algebraic geometry is concerned with *ideals* and their counterpart algebraic *varieties*. We give a very short description here. (Note that we shall use 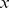 for variables in an abstract algebraic setting reserving 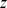 for “real” problems.)

A standard reference is [10] and in this and the next paragraph we present a very short summary of the first two chapters. We start with the ring of all polynomials in 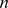 variables 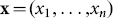 over a field *k*: 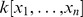. A set 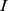 of polynomials in 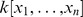, is an ideal if (i) zero is in 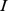, (ii) 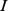 is preserved under addition and (iii) 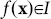 implies 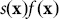 is in 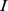 for any 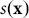 in 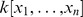. By a theorem of Hilbert all ideals are finitely generated. That is we can find a set of basis polynomials 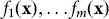 such that any 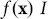 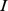 can be written 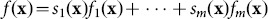 for some 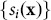 in 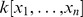. An ideal 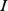 gives a variety as the set of 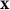 such that 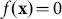 for all 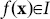. It will also be enough to work within the field 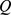 of rationals.

Modern computational algebra has benefitted hugely from the theory of Gröbner bases and the algorithms that grew out of the theory, notably the Buchburger algorithm. We will need one more concept, that of a monomial term ordering, or term ordering for short. Monomials 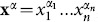, where 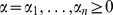 ie 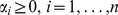, drive the theory. A monomial term ordering, written 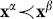 is (i) a total (linear) ordering with 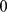 as the unique minimal element and the additional condition (ii) 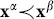 implies 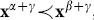, for all 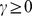. Since such an ordering is linear every polynomial 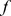 has a leading term with respect to the ordering: 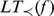. If we fix the monomial ordering, 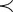, the Gröbner basis 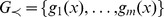 of an ideal 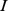 with respect to 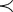 is a basis such that the ideal generated by *all* leading terms in the ideal is the same as that generated by the set of 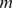 leading terms of the basis 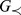. Given 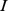 and 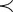 the Buchburger algorithm delivers 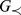. We will be concerned with the set of all Gröbner bases as 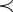 ranges over all monomial term orderings. This is called the *fan* and is finite, although it can be very large.

One of the main definitions of a toric ideal fits perfectly with the power product transformations of dimensional analysis. It is this observation which motivates this paper. We shall emphasize the connection by using the same notation: 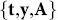, with 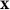 or 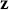 according to emphasis, in both the algebraic and physical theories.

The following development can be taken from a number of books, but [25] is our main source. The main steps in the definition are.

1. The polynomial ring over 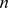 variables 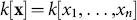.

2. A 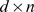 matrix 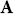 with columns labeled 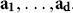.

3. Variables 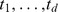 and the Laurent ring generated by the 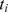 and the inverses 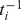. We write this as
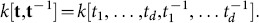



4. A power product mapping from 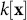 to 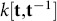 defined by 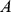:
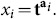



The kernel of the mapping in item 4 above is the toric ideal. It can be considered as the ideal obtained by formally eliminating the 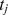 from the ideal:
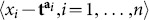



By formal elimination we mean in the algebraic sense as explained in [10], Chapter 3, that is obtaining the so-called elimination ideal: the intersection of the original ideal with the subring of polynomial excluding the 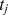.

The generators of the toric ideal are related to the kernel of 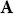 in the follow way. The generators are all so-called *binomials*

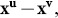
where 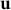 and 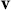 are non-negative integer vectors with the property that



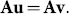



The last equation can be written 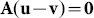, which is equivalent to 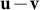 being in the kernel of 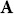.

The connection with dimensional analysis now becomes clear. Let us put dimensional analysis on a similar notational footing, only using 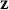 instead of 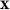. Start with a 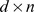 matrix 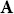 with columns 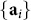. The general form of the mapping 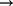 in (1), in the introduction, becomes
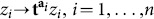
(2)


We can write this in matrix terms as
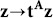
(3)


Now, suppose we have a possible invariant 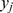. We first express 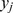 in terms of the 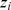. We use 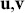 to denote integer vectors with non-negative entries to distinguish the positive from the negative exponents. Thus, we write
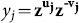



We are now in a position to test invariance. The necessary and sufficient condition for 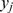 to be an invariant is that substituting each 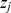 by 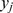 in the right hand side of (3) for 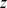 leaves 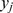 unchanged. But the condition for this is, replacing “
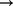
”, by “
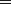
”
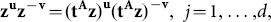



Cancelling the 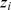 from both sides, this is equivalent to
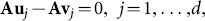
exactly the toric condition. We have proved our main result:

**Theorem 2.1** *A variable* 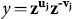 *, for non-negative integer vectors* 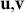 *is a dimensional invariant in a system defined by a matrix* 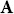 *, with derived variable* 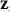 *, if and only if* 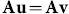 *. Moreover the set of all corresponding quantities*

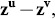

*form the toric ideal* 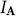 *with generator matrix* 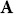.

A brief summary is that the set of all dimensionless variables 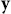 associated with a set of quantities defined by an integer matrix 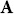 are exactly those given by the toric ideal 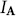.

We can give a minimal set of generators for the toric ideal of our running example. We use the Toric function on the computer algebra package CoCoA, [9], which takes the matrix 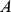 as input. Simply to ease the notation in the use of computer algebra we use 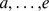, for 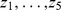. The script with output is.

Use 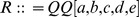;

Toric([[1,0,0,1,1], [−3,1,1,−1,1], [0,−1,0,−1,−2]]);

Ideal
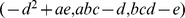


By the Theorem 2.1, given any generator we have an invariant. Thus 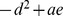 yields 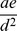 and we obtain three invariants:
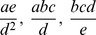



The second two terms give the dimensional variables from the alternative kernel matrix 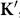, above. A key point is that the toric ideal may have more generators than the rank of the kernel in Buckingham's theorem. The next section amplifies this point.

### 2.1 Saturation and Gröbner bases

To summarise, the toric version of dimensional analysis says that we can generate dimensionless quantities from the toric ideal which is the elimination ideal of the original power product representation, being careful to use elimination in the proper algebraic sense.

A lattice ideal associated with an integer defining matrix 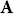 is the ideal based on a full rank kernel matrix. That is if 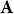 is 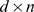 with rank 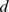 then we find an integer 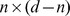 matrix 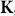, with rank 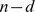 with rows 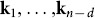 with 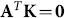.

The corresponding lattice ideal is generated by 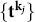. For our first 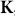 in subsection (1) *lattice ideal* has two generators:
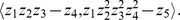



But, as we have seen, this is one fewer generators than the toric ideal. However, given any such lattice ideal we can obtain the toric ideal using a process called *saturation*. The process has two steps. Fix the defining matrix 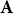 compute a kernel 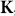 and let 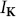 be a lattice ideal associated with 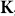.

1. Select a dummy variable 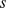 and adjoin to the lattice ideal the generator 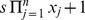. That is form the sum of the ideals:
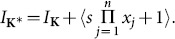



2. Eliminate 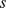 from 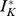 to give the toric ideal for 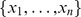. That is, the toric ideal is obtained as the elimination ideal for 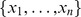.

The process of elimination in this saturation process is a formal procedure and leads to a *reduced* Gröbner basis of the toric ideal which depends in general on the monomial ordering used in the elimination algorithm. Reduced means all coefficients of the leading terms are 1 and removing basis terms gives a ring which cannot contained the remove term; essentially there is no redundancy.

Saturation gives an explanation for the fact that the toric ideal contains, but is not necessarily equal to the lattice ideal: the addition condition 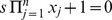 giving the variety defined by 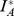 forces all the 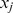 to be nonzero. Translated into the original 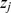 the toric ideal description of the dimensionless quantities has the physical interpretation that it gives the full set of rational polynomial invariants when *none of the defining variables* 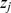 *is allowed to be zero*. This removal of zeros is intimately connected with the more abstract definitions of toric varieties but we do not develop this here, see [11].

## The Gröbner Fan, Primitive Invariants and the Graver Basis

A natural question, given the ease of computing invariants using toric methods, is whether the invariants obtained in this way are in some sense *minimal*. This turns out to be the case. We can illustrate this with our example. A little inspection of the basis 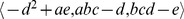 shows that we cannot get simpler invariants from this basis by multiplication (or division): if
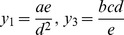
then 
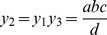
 which, although a new invariant, is not obtained by reducing the numerator or denominator of any of the original invariants.

**Definition 1** *A basis element* 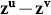 *of* 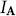 *is called is called primitive if there is no other (different) basis element invariant* 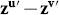 *such that such that* 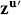 *divides* 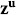 *and* 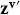 *divides* 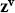 *. We call an invariant* 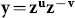 *primitive if and only if* 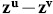 *is primitive as a basis element of* 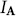 *.*

The following is straightforward alternative definition.

**Definition 2** *A dimensionless invariant* 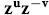 *is primitive if and only if it cannot be written as the product of two other such invariants which do not have common variables.*

In the above example 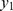 and 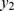 have 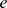 in common. In linear programming notation the condition is that there are no 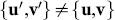 such that 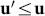 and 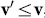, recalling that “
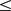
” means entrywise.

Lemma 4.6 of [25] is

**Lemma 3.1** *Every invariant obtained from a reduced Gröbner basis of* 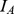 *is primitive.*

Note that in what follows we are a little lazy in not to distinguish an invariant from its inverse.

As mentioned, as we range over all monomial term orderings defining the individual Gröbner basis we obtain the complete Gröbner fan and by the Lemma 3.1 and our definition all resulting invariants are primitive. This union of bases is called the *universal* Gröbner basis and the computer programme Gfan is recommended to compute the fan [20].

We return to our running example. If we put the G-basis element 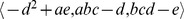 into Gfan we obtain the full fan as
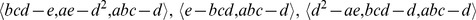


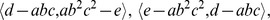
the first of which is the input basis.

The universal Gröbner basis of distinct basis terms (ignoring the sign change) is:
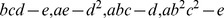
and we have a new primitive invariant, namely our 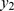 in the introduction.

**Definition 3** *The set of all primitive basis elements (which may be larger than the universal Gröbner basis), is called the Graver basis.*

Algorithm 7.2 of [25] can be used to compute the Graver basis. The method starts by constructing from 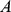 an extended matrix called the Lawrence lifting:
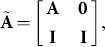
where the zero is a 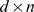 zero matrix and 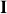 is a 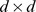 identity matrix. Then introducing 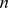 more derived variables to make a set 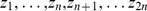 a toric ideal is constructed using 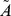. Finally, set 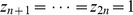.

The method is conveniently set out in the help screen of “ToricIdealBasis” on Maple. After inputting
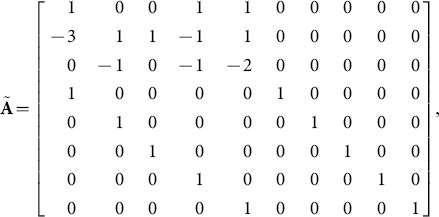
we use the Maple command



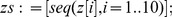


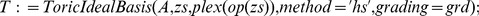


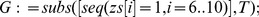



This yields
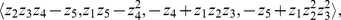



In this case the set is the same as given by the fan. That is, the universal Gröbner basis and the Graver basis are the same.

## Further Examples

For each of the examples below we give the derived quantities using the classical notation, (i) the 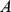 matrix (ii) a single toric ideal basis given by the default function on CoCoA and (iii) a full set of primitive basis elements, that is the Graver basis, given by the maple ToricIdealBasis command and the Lawrence lifting. From this a full set of primitive invariants is immediate. It turns out that for all except one of our examples (windmill) the Graver basis is also the universal Gröbner basis. We mention when we find well-known invariants.

### 4.1 Windmill

This standard problem is taken from [15], Section 9.3. (We have changed 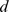 there to 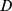).

It concerns a simple windmill widely used to pump water. The units are: is

shaft power, 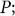 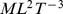


diameter, 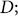 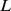


wind speed, 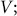 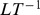


rotational speed, 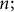 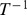


air density 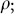 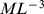


The 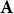-matrix is
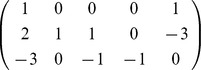



In the 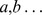 notation we obtain, from CoCoA, a basis with 4 terms:
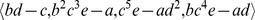



The first entry give a dimensionless quantities discussed in the book:
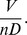



The universal Gröbner basis obtained from the Gfan gives five terms
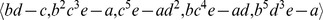



The last of these is also discussed in the book; it gives the invariant
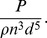



A full set of 7 primitive invariants, the Graver basis, is
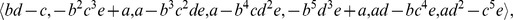



Since 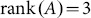 there are only two algebraically independent invariants. The standard argument may suggest testing the relationship between any two independent invariants, for example in a wind tunnel. An important question, which should be the subject of further research, is say which two or, more generally, whether the dimensional analysis is sufficiently trusted to test only one pair and infer other relationships from the algebra.

### 4.2 Forced convection

The interest is in the following derived quantities: the forced convection coefficient 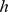, the velocity, 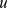, the characteristic length of the heat transfer surface 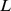, the conductivity of the fluid 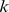, the dynamic viscosity, 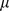, the fluid specific heat capacity, 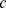 and the fluid density, 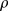. The fundamental dimensions are 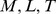 and two new ones temperature (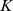) and energy (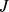). The table of units is

forced convection coefficient, 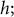 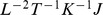


velocity, 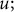 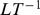


heat transfer surface length, 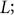 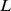


thermal conductivity of the fluid, 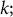 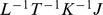


fluid dynamic viscosity, 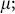 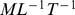


fluid specific heat capacity, 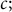 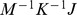


fluid density, 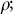 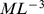


With columns in the order of the listed the rows in the units order the 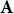-matrix is
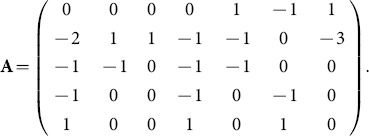



Resorting to the 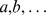 notation we have from, CoCoA, the ideal
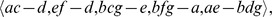
giving invariants:



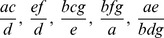



The first three of these are well-known invariants:
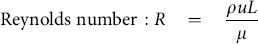
(4)

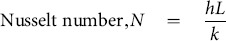
(5)

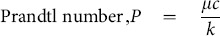
(6)


In preparing this paper it was pleasing to obtain these directly from the computer on the first run. The full set of 7 primitive basis elements is
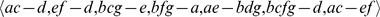



The simplest of the “new” primitive invariants is from 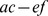:
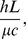
which is the Reynolds number divided by the Nusselt number.

### 4.3 Electrodynamics

As an exercise we take six basic quantities for electro-dynamics and use the literature to give some expression in terms of mass 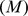, length 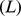, time 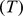 and current (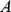). We do not have any particular electromagnetic device in mind, but simply try to find some dimensionless quantities. The following is one version:

charge 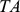


potential 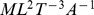


capacitance 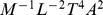


inductance 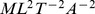


resistance 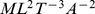


Then
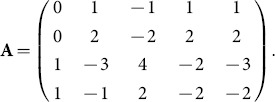



CoCoA gives
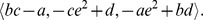



Note that 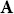 only has rank 3. It turns out that this is a complete list of primitive basis elements.

### 4.4 Hydrogen atom

Toric ideals are embedded in advanced models in physics but one can get some way with simple dimensional analysis. This example is given in some form by a number of authors. We found [24], section 1.3.1, useful. The hydrogen atom consists of a proton and a neutron and the Bohr radius is the distance between them. We have used slightly non-standard notation. In a somewhat cavalier manner we have introduced the speed of light as a derived quantity.

mass of electron, 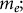 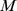


Bohr radius, 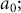 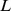


energy, 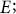 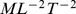


Plank's constant, 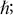 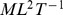



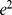
 (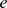 is elementary charge); 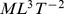


speed of light, 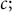 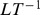


Then
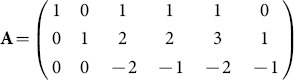



The ideal is
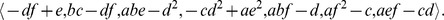



(The algebraic 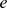 is 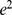 and the algebraic 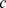 should not be confused with the speed of light). The first terms gives an invariant called the “fine structure constant”
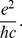



If we take the third term and interpret 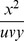 being invariant as stating that 
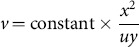
, then we have a well known formula for 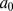 interpreted as the size of the hydrogen atom:
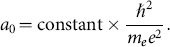



We cannot resist stating that the sixth basis element, 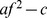 gives
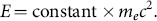



The Graver basis gives a set of 10 primitive invariants for the hydrogen atom:
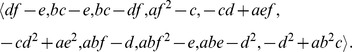



It is not known whether this list has been given explicitly before.

## The Wider Picture: Group Invariance

Dimensional analysis should be considered as a special case of the theory of group invariance and in an attempt to suggest a natural generalisation we very briefly sketch the theory of invariants.

We start with the action of a Lie group 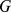 acting on a manifold 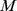 in 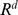. The manifold will be our model and the group something to do with our physical understanding. The *orbit* of 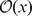 for a point 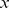 in 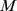 be the set of all 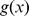 for all 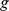 in 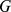. If 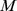 is invariant under 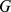 then 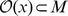. This sets up an equivalence relation with members of 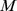 in which the same orbit are equivalent. The collection of equivalence classes is denoted by the quotient 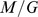 and the *projection* 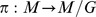 maps every member of of 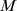 into its correct equivalence class. Under suitable conditions 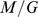 is a manifold in its own right and we say that 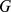 acts *regularly* on 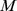. Also, the mapping 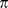 can be used to set up a coordinate system on 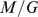 and note that 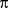 itself is an invariant. This discussion leads naturally to the following.

**Proposition 5.1** *Let a group* 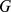 *act regularly on a manifold M. A manifold defined by a smooth function* 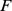 *is a set* 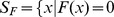 *. It is* 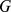 *-invariant if and only if there is a function* 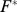 *defining a smooth sub-manifold* 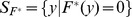 *on* 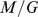 *such that*

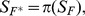

*where* 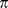 *is the projection from* 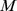 to 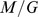.

A one parameter Lie group 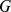 shifts a point 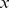 along an integral curve 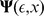 by a *flow*. If we expand 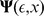 in a Taylor expansion in 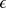 we obtain:
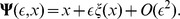



The term 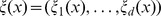 defines a vector field and we can write 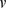 in classical local coordinates:
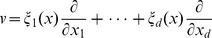



A function 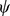 is an invariant if 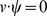, namely
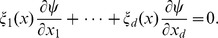



This is a first order partial differential equation which can be solved by first writing down
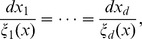
namely by the methods of characteristics. The solutions take the form:



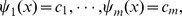
where the 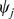 are the invariants.

In our notation 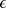 becomes 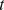 and using our generating matrix 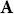 the mapping 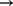 in (3) is
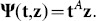



Performing matrix partial differentiation with respect to 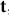, and setting all 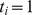 gives the infinitesmal generators:
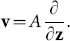



An interpretation of the toric variety is as characterising the orbits of the scale group of transformation, as discussed above. We have not formally proved the Buckingham theorem, but drawing on the above discussion it is given as Theorem 2.22 in [23].

## Limitations of the Study, Open Questions and Future Work

We have seen that the toric ideal method, via the Graver basis, is a fast way to compute all primitive invariants in dimensional analysis. There are some areas of further study which this suggests.

The first area arises from the possibility that different physical systems may yield different types of toric ideal or variety. The most important general class is *normal* toric varieties. Briefly, such varieties are related to polyhedral cones and polyhedra with integer or rational generators. The standard approach is to take the a suitable cone 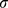 and compute its Hilbert basis, which is a set of integer generators of the dual cone giving all integer grid points in that cone. From this there is a natural toric ideal. But an open problem, it seems to the authors, is whether this rich theory of normal varieties and polyhedra has a role in classical physics and engineering.

A second area is a natural development from the previous section. A discussion missing from this paper is the way in which differentials are converted to derived quantities. For example velocity, which is 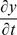, for some length variable 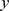 and time 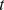 is allocated the units 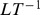. One way to keep the advantages of awarding derived quantities to differential terms, but retain differentials is to use combinations of differential and polynomial operators. The algebraic environment which allows this is differential algebra and in particular Weyl algebras. Much of the existing work uses the methods to study identifiability (see [22]) but the challenge, here, is to find differential-algebraic invariants in the same spirit as the invariants in this paper. A noteworthy development is [17]–[19], especially in the context of dynamical systems.

Finally, the authors are aware of the value of standard dimensional analysis when the exponents are rational but not necessarily integer. This arises in the work on turbulence mentioned [7], [8]. This can be studied by changing the base lattice to have fractional levels and the authors are considering research in this area.

## Conclusions

Toric ideals are the appropriate framework for dimensional analysis with describe the rational invariants under the multivariate scale group. The key contribution is that the Graver basis, which can be the thought of as a unique “envelope” of bases for the toric ideal and consists of all primitive elements then gives rise to a notion of primitive invariants. These comprise the invariants which cannot be split into the product of two other invariants using *disjoint* sets of units. The suggestion is that this solves a classical but sometimes unspoken conundrum in dimensional analysis: namely that invariants produced by the celebrated Buckingham method are not unique. In addition the Graver basis is easily computed using computer algebra. In some examples we are able to find some named invariants, all, satisfyingly, primitive. But also, because the Graver basis is larger than a minimal basis for the toric ideal the methods throws up new primitive invariants which may attract scientific interest.
